# Hip dislocation with ipsilateral shaft of femur fracture: A rare case report

**DOI:** 10.1016/j.ijscr.2025.111674

**Published:** 2025-07-16

**Authors:** Ratish Singh, Amit Ranjan Mishra, Hem Shankar Yadav, Hridhika Yadav

**Affiliations:** Janaki Medical College and Teaching Hospital, Nepal

**Keywords:** Avascular necrosis, Advanced trauma life support, Case report, Hip dislocation, Shaft of femur fracture

## Abstract

**Introduction:**

Posterior hip dislocation with ipsilateral shaft of femur fracture is a very rare case encountered. This case presents a rare combination of posterior hip dislocation with ipsilateral femoral shaft fracture with head of femur fracture of a sixteen year old male patient following a road traffic accident (RTA). It emphasizes the importance of a multidisciplinary trauma protocol, early imaging, and timely surgical intervention in complex orthopedic injuries.

**Case presentation:**

Patient was presented in the emergency department with complaints of severe pain and deformity in the left hip and thigh, inability to bear weight, and laceration below the left knee. After initial emergency management patient was operated on with closed reduction of the hip and intramedullary interlocking nailing of the femur after 6 h. Postoperatively, skin traction was applied for 2 weeks to prevent re-dislocation of the hip. Successful surgical management with good functional recovery was achieved. At 2 weeks, the patient was ambulatory with non-weight bearing and crutch walking with no complications seen.

**Clinical discussion:**

This case underscores the need for protocol-driven, multidisciplinary care in adolescent trauma, with emphasis on early intervention and comprehensive assessment for preventing the patient from complications such as avascular necrosis of the femoral head.

**Conclusion:**

Posterior dislocation of the hip with ipsilateral shaft of femur fracture is an uncommon type of injury where early emergency management followed by prompt surgical intervention is required to prevent avascular necrosis of the femur head. Regular follow-up is essential for monitoring and assessment of condition of hip.

## Highlights

• Posterior dislocation of the hip with ipsilateral shaft of femur is a rare injury typically resulting from high-energy trauma.
• The condition requires a multidisciplinary approach, and we managed the patient under ATLS protocol and scheduled surgery as early as possible.
• Early intervention and prompt surgical intervention (within 6 h) lowers the risk of avascular necrosis of the femoral head.
• A temporary external fixator was applied to align the fracture and facilitate closed reduction of the dislocated hip using the Allis maneuver.
• Definitive fixation of the femur shaft was done with intramedullary interlocking nailing, leading to good alignment and stability.
• Post-operatively, by day 14, the patient was mobilized with non-weight-bearing ambulation under physiotherapy and was discharged without complications.

## 1 Introduction

Posterior hip dislocation with an ipsilateral shaft of femur fracture is a rare injury typically occurring due to high-energy trauma such as motor vehicle accidents or falls. The force is applied to the proximal fracture fragment, which is preceded by the dislocation of the hip [1,2]. Early implementation of Advanced Trauma Life Support (ATLS) protocols in patients with hip dislocation is the cornerstone of trauma care, ensuring the airway, breathing, and circulation before conducting any other examinations [3]. After the patient is stabilized, thorough physical examination of the affected limb may reveal the attitude of flexion, internal rotation, adduction, and appear shorter than the contralateral limb [4].

**Fig. 1:**
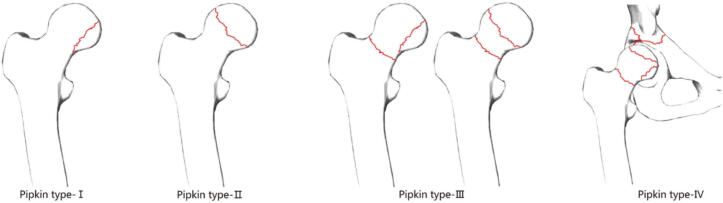
Pipkin classification of femoral head fractures with posterior hip dislocations. (Reproduced from Zhang et al. (2023), Journal of Orthopedic Surgery and Research, under the Creative Commons Attribution 4.0 License.) [8].

The diagnosis of hip dislocation may be masked as the focus is more towards the apparent shaft of the femur fracture [5]. The most commonly used classification in posterior hip dislocation is Pipkin classification, which is a subset of the Thompson and Epstein classification [6,7]. Pipkin types I and II are distinguished by the position of the fracture in relation to the fovea. Type I is below the fovea with the fracture outside of the weight-bearing joint parts, whereas type II fractures involve the more cranial, weight-bearing parts. Type III is any fracture of the head in combination with a femoral neck fracture. Additional fractures of the acetabulum are classified as type IV (Fig. 1) [8]. Nevertheless, some complex fracture patterns and posterior hip dislocation linked to ipsilateral shaft of femur fracture are not included in these classifications.

Early surgical management is required in such cases and commonly performed surgical techniques include manual closed reduction and operative fixation. The problem may arise due to difficulty in aligning the fracture segment or due to insufficient traction, which can make it challenging to achieve a proper closed reduction [9,10]. In such situations, operative fixation becomes the preferred approach to ensure stability, promote proper healing, and restore anatomical alignment. Distinct fixation methods are applied depending upon the nature and severity of the fracture, most commonly intramedullary nailing is done for close shaft of femur fracture. Early intervention, ideally within 6 h, reduces the chances of avascular necrosis (AVN) of the femoral head [2,10]. For posterior hip dislocations, the event rate for AVN ranges from 10.6 % to 43.0 % [11]. We present a case of a sixteen year old boy who met with a motor vehicle accident and presented to the emergency department. After initial management, radiodiagnosis confirmed the case of posterior hip dislocation with ipsilateral shaft of femur fracture. This case report was held in line with the Surgical Case Report (SCARE) checklist [12].

## 2 Case presentation

A sixteen years old male involved in a motor vehicle collision was brought via ambulance to the emergency department (ER) of Janaki Medical College and Teaching Hospital (JMCTH). On presentation, he had severe pain over the left hip region with bleeding below the knee. Initially, he was managed according to the Advanced Trauma Life Support (ATLS) protocol, where the airway was protected and normal respiratory effort was observed. Bleeding was controlled in the ER along with the immediate initiation of intravenous (IV) fluids. The Glasgow Coma Scale (GCS) was recorded as 14/15. A secondary survey was completed, which revealed no physiological alteration at that time. A focused assessment with sonography in trauma (FAST) scan was performed and yielded negative results.

The patient experienced severe pain in the hip region, with a Visual Analog Scale (VAS) score of 8/10, which prevented us from performing a physical examination and range of motion (ROM) assessment of the hip. The attitude of the limb was flexion, adduction, and internal rotation. There was swelling around the thigh and knee joint, along with abrasions below the knee joint. Distal neuro-vascular status (DNVS) was assessed, which was intact.

Upon taking a proper history after stabilization, there was no prior medical or surgical history, no known allergies, no family history of orthopedic or genetic conditions, and no comorbidities. We ordered lab investigations such as complete blood count (CBC) the results of which are shown in (Table 1), serology and basic biochemistry, results in (Table 2). The computed tomography (CT) scan was planned initially, but could not be performed due to temporary unavailability of the CT scanner at our institution as a result of equipment malfunction.

**Table 1:** Results of CBC Test.

S. No	Test	Result	Unit	Reference Range
1.	Total Leucocyte Count	16,460	Per μl	5000–11,000
2.	Differential Leukocytes Count			
	Neutrophil	85	%	40–75
	Lymphocyte	10	%	20–45
	Eosinophile	00	%	1–6
	Monocyte	05	%	2–10
	Basophil	00	%	0–1
3.	Total RBC Count	4.18 × 10^6^	/μl	4.5–5.5
4.	Hemoglobin	12.0	gm/dl	14–16
5.	Hematocrit	37.5	%	40–50
6.	MCV	89.8	fl	76–96
7.	MCH	28.7	Pg	27–32
8.	MCHC	32.0	gm/dl	31.5–34.5
9.	RDW-CV	15.6	%	11.5–15.5
10.	RDW-SD	45.6		–
11.	Total Platelet Count	160 × 10^3^	/μl	150–400

**Table 2:** Results of serology and basic biochemistry.

S. No.	Test	Result	Unit	Reference Range
1	Blood Group & Rh Type	B Positive	–	–
2	HIV I & II	Negative	–	Negative/Positive
3	HBsAg	Negative	–	Negative/Positive
4	HCV	Negative	–	Negative/Positive
5	Sodium	136.9	mmol/L	135–145
6	Potassium	4.3	mmol/L	3.5–6.0
7	Blood Urea	25.1	mg/dl	15–45
8	Serum Creatinine	1.0	mg/dl	0.6–1.2

**Fig. 2:**
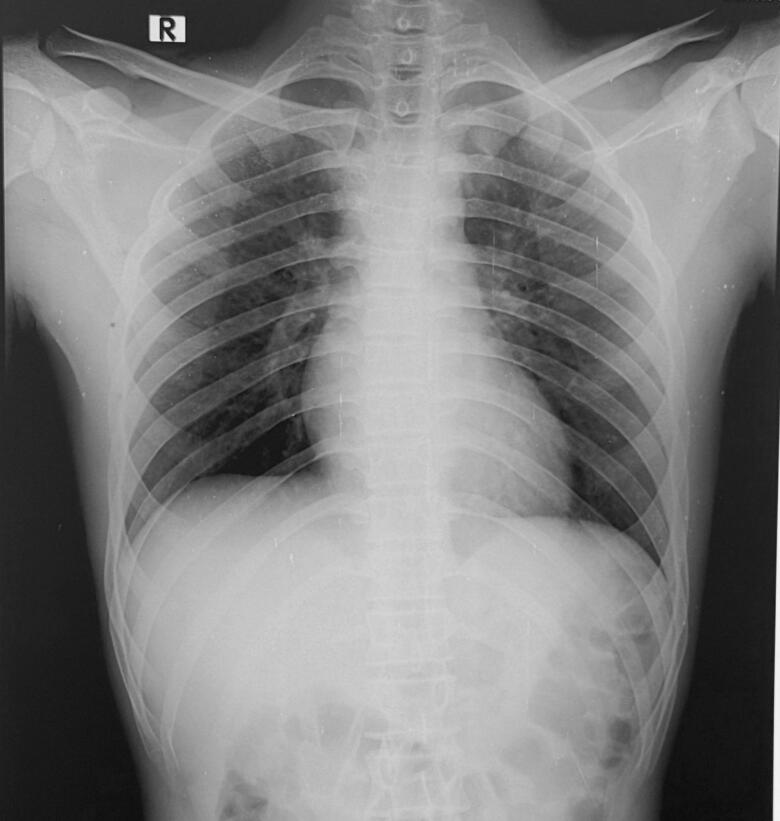
Chest x-ray showing lung fields clear bilaterally with no evidence of ribs fractures or pneumothorax. The cardiac silhouette and mediastinum appear within normal limits.

**Fig. 3:**
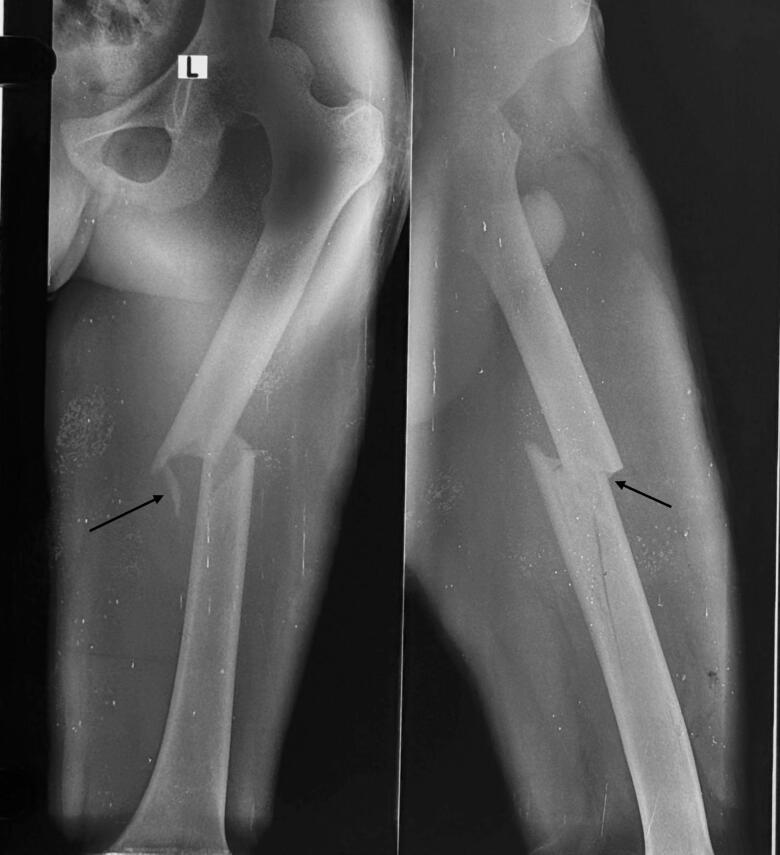
Pre-operative anteroposterior and lateral x-ray of femur showing mid-shaft femur fracture with a small fragment.

**Fig. 4:**
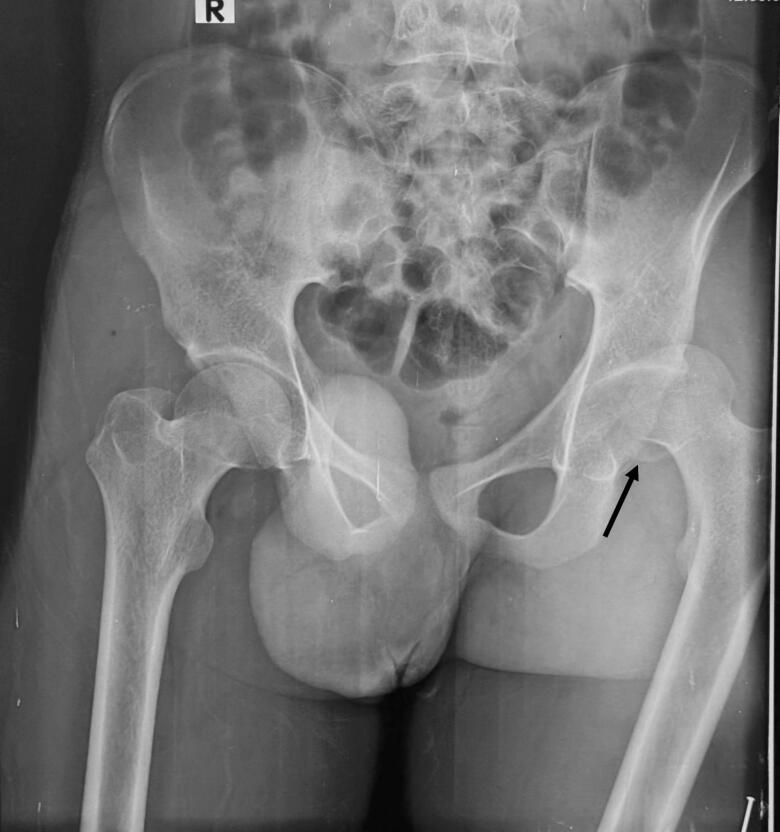
Pre-operative x-ray of pelvis with bilateral hip joint showing posterior dislocation of left hip with femoral head fracture confined to the inferior portion of the femoral head appearing as small, isolated fragment detached from the femoral head.

**Fig. 5:**
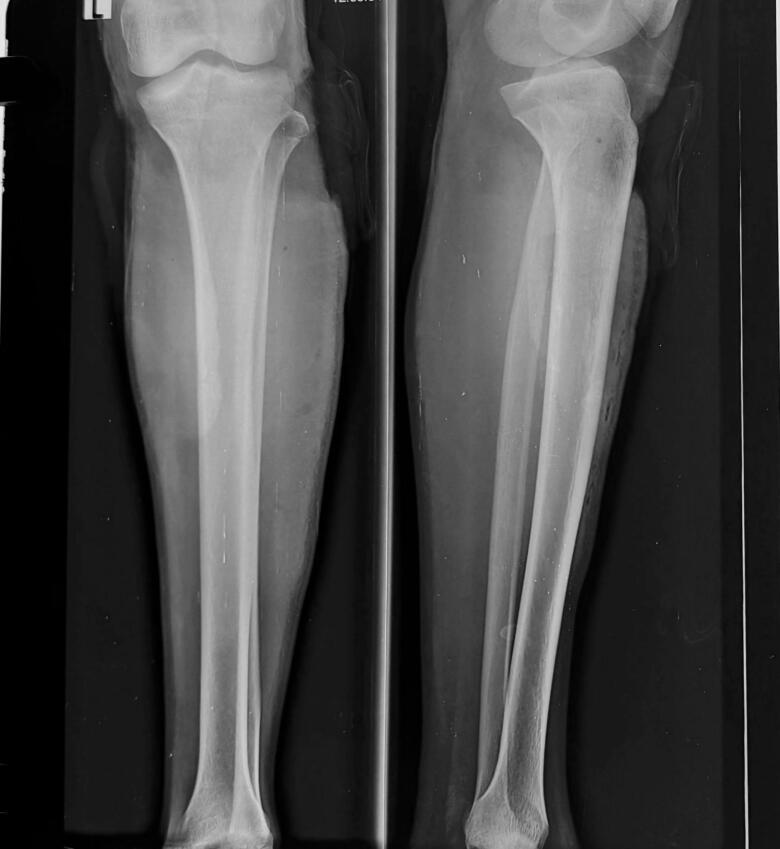
Pre-operative x-ray leg anteroposterior and lateral view shows no evidence of any fractures.

Chest X-Ray revealed lung fields clear bilaterally with no evidence of any ribs fracture or pneumothorax (Fig. 2), anteroposterior (AP) and lateral X-Ray of the femur revealed a mid-shaft fracture of the femur (Fig. 3), X-ray pelvis revealed posterior dislocation of the left hip joint along with femoral head fracture which is confined to the inferior portion of the femoral head and appearing as small, isolated fragment detached from the femoral head (Fig. 4). This injury pattern is consistent with a Pipkin Type I femoral head fracture (Fig. 1). There was no radiological evidence of femoral neck fracture or associated acetabular wall involvement, ruling out Pipkin Types III and IV respectively. The diagnosis was made based on standard anteroposterior pelvic radiography in the absence of CT imaging. AP and lateral X-Ray left leg showed no evidence of fracture (Fig. 5).

## 3 Timeline

The chronology of the events is presented in Table 3.

**Table 3:** Timeline of events in chronological order.

Event	Description
Day 0 (Day of Injury)	RTA and ER admission, ATLS protocol initiated, initial imaging, and blood tests were performed
Day 0 (Day of Injury)	Underwent closed reduction of hip dislocation and internal fixation of femoral fracture after 6 h of initial management.
Day 1	A transfusion of 1 pint (i.e. 473.18 mL) of whole blood was performed, and skin traction was applied.
Day 2	Transfusion of 1 pint (i.e. 473.18 mL) of whole blood was done.
Day 3	The patient developed abdominal pain, and CT abdomen revealed a peritoneal collection, which was managed conservatively. Operative incision site dressing was done. Foley's Catheter was removed.
Day 14	Repeat X-ray of the pelvis was done. Skin traction was removed. Mobilized non-weight-bearing under Physiotherapist Supervision.
Day 16	The patient was discharged from the hospital with no complications noticed during the stay. Counselling was done with advice for follow-up after 2 weeks.

## 4 Therapeutic intervention

Operative intervention was planned after 6 h of stabilization of the patient after injury. Blood transfusion was not preferred before surgery because the patient was hemodynamically stable, as well as the laboratory investigation report was also within normal limits. However, one pint (i.e. 473.18 mL) of blood was arranged for the intra-operative period incase required. The surgery was done under epidural anesthesia because the procedure was expected to be prolonged. Closed relocation was attempted after, but unfortunately, it was not successful without stabilizing the fracture site, i.e. shaft of the femur. Thereafter, an external fixator (Ex-Fix) was applied under C-ARM guidance to make the fracture site stable and to convert it into a single fragment so as to make the traction and counter traction easier. After that we attempted to relocate the dislocated hip joint and finally it was relocated using the ALLIS maneuver. All the procedure was performed with the patient in supine position. We didn't try to fix the femur head since post-relocation the fractured fragment showed satisfactory alignment. The fracture was classified as Pipkin Type-I **(**Fig. 1**)**. Relocation was confirmed intraoperatively with C-ARM imaging.

**Fig. 6:**
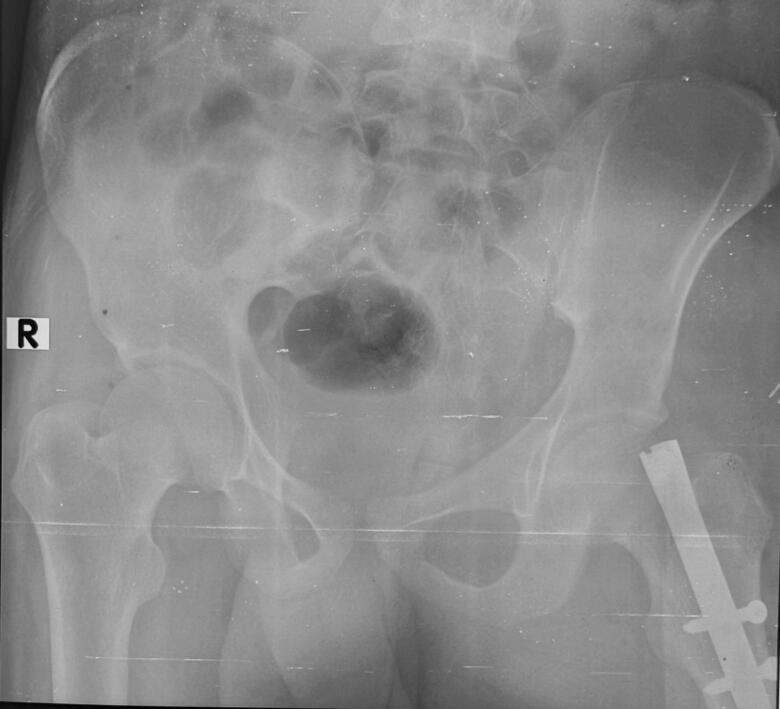
Post-operative x-ray pelvis with bilateral hip joint showing reduction of left hip.

**Fig. 7:**
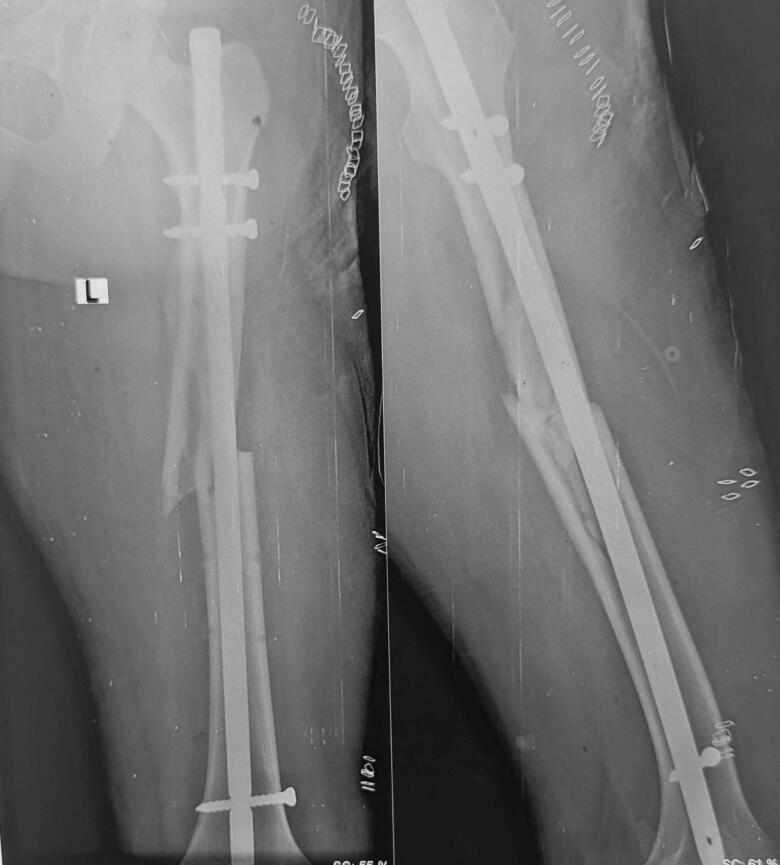
Post-operative x-ray left femur full length anteroposterior and lateral view shows intramedullary femoral nail in situ.

After that the patient was kept in lateral position for definitive femoral shaft fixation. We didn't use traction table for operating shaft of femur fracture. A lateral incision around 7 cm was made superior to greater trochanter region of left femur. Piriformis entry was done for the insertion of the entry point using awl. Guide wire insertion was done after taking out the external fixator and maintaining the reduction as it was done before. Intramedullary rimming was done with cannulated rimmer from 8 mm to 11 mm. Finally, a 10 × 36 mm intramedullary nail was kept with distal single screw and proximal static and dynamic screw. Maintaining hemostatic condition closure was done in layers. Post-operatively the X-ray of pelvis was done which showed bilateral hip joint with reduction of left hip (Fig. 6) also X-ray of fracture site i.e. shaft of femur seemed to be extended proximally due to manipulation done after external fixator (Fig. 7) We didn't use traction table for operating shaft of femur fracture. After reduction of hip, we operated the shaft of femur in lateral position since already alignment was achieved at the time of external fixator. Therefore, no need of further using traction table as a result, intra operative time was also minimized.

Post-operatively, the Foleys catheter was kept for 3 days and then it was removed. Wound dressing was performed at 2 days intervals. No any superficial infection around the wound was seen which noted the area to be healthy. One pint (i.e. 473.18 mL) of blood each day was transfused on post-operative day 1 and 2. On day 14th, sutures were removed along with discontinuation of skin traction (Fig. 9) and a pelvic X-ray was obtained to assess the location of hip (Fig. 10). Mobilization was started under physiotherapist supervision. Strict non-weight bearing and crutch walk was advised to the patient. After rehabilitation education provided to the patient, then after patient was discharged.

**Fig. 8:**
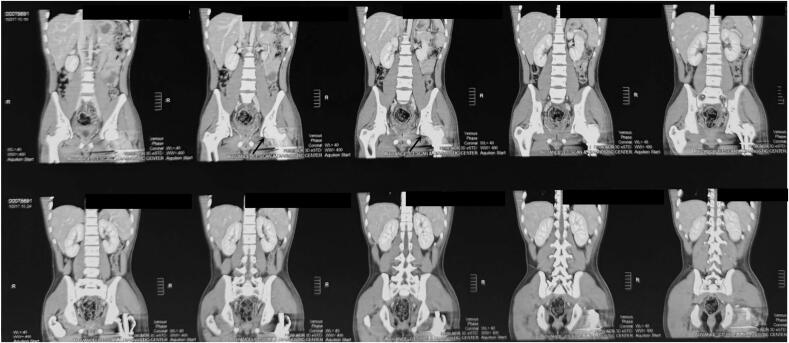
Post-operative CT scan of the pelvis and hip with linear, longitudinal and non-displaced fracture of head of left femur at the level of fovea capitis (Pipkin Type 1) marked by arrows.

Revision USG was done after 2 days of surgery since the patient complained of severe abdominal pain. Minimal fluid collection was seen on USG. Thereafter CT scan of the Abdomen/Pelvis was advised by the general surgeon to rule out other pathology, which revealed minimal intraperitoneal collection in the pelvic cavity and graded as per Adapted Clavien-Dindo in trauma (ACDiT) i.e. grade 1 [13]. Also, slices of pelvis and hip on the CT scan showed linear, longitudinal and non-displaced fracture i.e. fracture outside of the weight-bearing joint below the level of fovea (Pipkin type I) **(**Fig. 8**)**. Conservative management for the intraperitoneal collection was done after the CT diagnosis of the pelvis and abdomen.

**Fig. 9:**
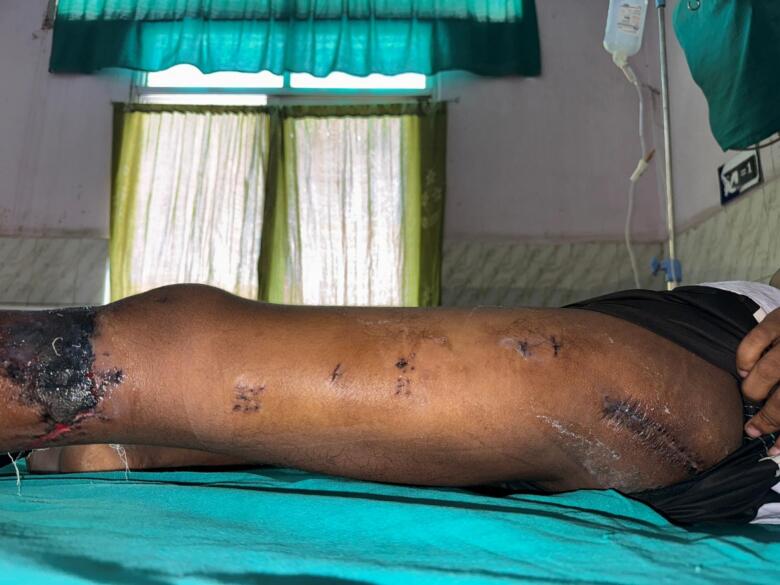
Post-operative day 14 -suture removal and discontinuation of skin traction.

**Fig. 10:**
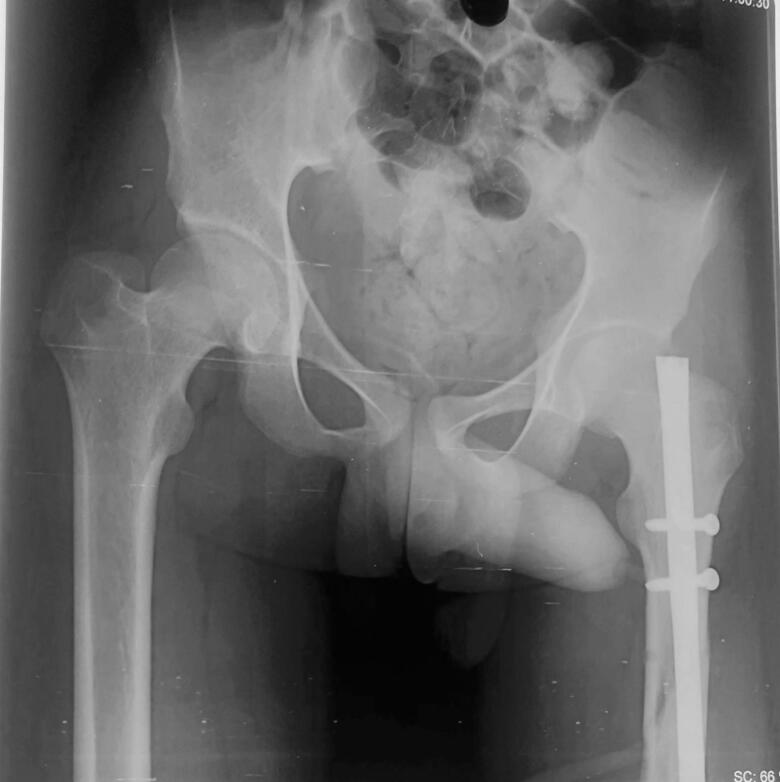
Post-operative day 14 x-ray pelvis with bilateral hip satisfactory.

## 5 Discussion

This case describes a sixteen year old boy who was involved in an accident and suffered a rare combination of a femur head fracture classified as Pipkin type I, left posterior dislocation of hip with ipsilateral shaft of femur fracture. It is a complex and rare pattern of injury typically occurring due to high energy trauma. This simultaneous occurrence complicates the diagnosis and management, which often leads to delayed treatment and increased risks of complications. It is an emergency situation that requires prompt reduction (usually within 6 h) once the patient is hemodynamically stable; elsewise, there is a high risk of the femoral head going into avascular necrosis [2,10]. In our case we made the diagnosis on the basis of X-ray due to the unavailability of a CT scan. After managing the patient based on ATLS protocol, closed reduction of the left hip was done after stabilizing the shaft of the femur with external fixator. Definitive fixation was done using intramedullary nailing. Post-operative CT scan confirmed Pipkin type I fracture, and the patient recovered well with conservative management for mild abdominal fluid collection which was managed conservatively. Long-term follow-up is essential to detect possible complications such as avascular necrosis, and the patient should be monitored periodically with radiographical imaging. We have scheduled the follow up for 3, 6, and 12-month in our case.

Similar case was managed using a temporary external fixator applied to the proximal femoral shaft, which served as a handle to facilitate closed reduction of the hip dislocation, which was then followed by intramedullary nailing of the femoral shaft [14]. This method allowed the orthopedic surgeons for closed reduction without requiring open surgery, thereby reducing the risk of open reduction complications. In our case, we took a similar technique, putting a temporary external fixator to the proximal femoral shaft to permit closure reduction of a posterior hip dislocation. We then performed intramedullary nailing of the femoral shaft fracture. This method enabled the efficient treatment of both injuries in a single surgical session, avoiding the requirement for extended surgical exposure and the risk of complications.

Another similar case was managed by Iftekhar et al. where they achieved the proper leverage and reduction by closed reduction and intramedullary nail fixation in conjunction with a Schanz screw [15]. The conservative management of Pipkin I femoral head fracture was done since after relocation of hip joint, the anatomical reduction of femoral head was also achieved. Other study as well shows that conservative management for type I femoral head has also good functional outcome in future [16].

If closed reduction is not successful, immediate conversion to open reduction and internal fixation (ORIF) is recommended [14,17]. Some cases reported the same as they required open reduction due to irreducibility by closed methods like Jadib et al. reported a case of irreducible posterior fracture-dislocation of the hip with ipsilateral shaft of femur fracture, where the head of femur had crossed over a posterior acetabular wall fragment which required open reduction and internal fixation [5]. Similarly, Sharma et al. reported a case managed with open reduction of hip and fixation of neck of femur with screws with distal femoral nail (DFN) for shaft of femur with good functional outcome and with no avascular necrosis of femoral head after 2 years of follow up [18]. After ORIF, complications such as bleeding, fracture loss, infection, avascular necrosis of the femur head, delayed union, and damage to the sciatic nerve can occur [19]. We managed this case without any serious postoperative surgical complications; education was provided before discharging and have advised the patient for regular follow-up.

## 6 Conclusion

This case report shows that there are no serious surgical or postoperative complications with early intervention in ipsilateral shaft of femur fracture, accompanied by posterior hip dislocation. Although no immediate complications were noted during the early postoperative period, longer-term follow-up is necessary to monitor for potential late complications including AVN. At the 2-week follow-up, there was no radiological evidence of AVN; however, further follow-up is necessary to rule out complications. The patient should be monitored in follow-up for 3, 6, and 12-month with radiographic imaging to access the condition of hip.

## Consent

Written informed consent was obtained from the patient's parents/legal guardian for publication and any accompanying images. A copy of the written consent is available for review by the Editor-in-Chief of this journal on request.

## Ethical approval

Ethical review and approval were not required for the case report since the university waives ethical approval. The case report doesn't contain any personal information of the patient.

There is a requirement of consent before using images of the patient, which was obtained from the patient's guardian and is available for review by the Editor-in-Chief of this journal on request.

Name of the University: Tribhuvan University.

Name of College: Janaki Medical College and Teaching Hospital.

## Sources of funding

The author(s) received no financial support for the research, authorship, and/or publication of this article.

## Author contribution

Ratish Singh: Treating physician, supervision, and review.

Amit Ranjan Mishra: Treating physician, supervision, and review.

Hem Shankar Yadav: Concept and writing- review and editing.

Hridhika Yadav: Conclusion and image.

## Guarantor

Hem Shankar Yadav accepts full responsibility for the work and/or the conduct of the study, had access to the data, and controls the decision to publish.

## Research registration

None.

## Declaration of Generative AI and AI-assisted technologies in the writing process

During the preparation of this work the author(s) used Grammarly in order to enhance language, grammar, and clarity. After using this tool/service, the author(s) reviewed and edited the content as needed and take(s) full responsibility for the content of the publication.

## Conflicts of interest

The authors declare no conflict of interest.
